# Supplementary material for: Modeling glioblastoma heterogeneity as a dynamic network of cell states
Source: Mol Syst Biol. 2021 Sep 16;17(9):e10105. doi: 10.15252/msb.202010105 (PMC8444284; doi:10.15252/msb.202010105)
Supplement: Supplementary file 5 — Source Data for Figure 3 [file MSB-17-e10105-s001.zip › Figure3A_sourcedata/GSEA_3065/nosigdb_state1.GseaPreranked.1622718409535/LARSSON_STATE_1.html]

Details for gene set LARSSON\_STATE\_1[GSEA]

|  || Dataset | state1 |
| Phenotype | NoPhenotypeAvailable |
| Upregulated in class | na\_pos |
| GeneSet | LARSSON\_STATE\_1 |
| Enrichment Score (ES) | 1.0000002 |
| Normalized Enrichment Score (NES) | 3.6068785 |
| Nominal p-value | 0.0 |
| FDR q-value | 0.0 |
| FWER p-Value | 0.0 |
Table: GSEA Results Summary

  

Fig 1: Enrichment plot: LARSSON\_STATE\_1      
 Profile of the Running ES Score & Positions of GeneSet Members on the Rank Ordered List

  

| PROBE | GENE SYMBOL | GENE\_TITLE | RANK IN GENE LIST | RANK METRIC SCORE | RUNNING ES | CORE ENRICHMENT || 1 | CCK |  |  | 0 | 0.961 | 0.0186 | Yes |
| 2 | S100A6 |  |  | 1 | 0.884 | 0.0358 | Yes |
| 3 | TMSB4X |  |  | 2 | 0.859 | 0.0525 | Yes |
| 4 | TMEM158 |  |  | 3 | 0.835 | 0.0687 | Yes |
| 5 | LGALS1 |  |  | 4 | 0.828 | 0.0847 | Yes |
| 6 | SCG2 |  |  | 5 | 0.825 | 0.1007 | Yes |
| 7 | GCHFR |  |  | 6 | 0.778 | 0.1158 | Yes |
| 8 | ODC1 |  |  | 7 | 0.744 | 0.1302 | Yes |
| 9 | HMGA1 |  |  | 8 | 0.736 | 0.1445 | Yes |
| 10 | LMNA |  |  | 9 | 0.701 | 0.1581 | Yes |
| 11 | NQO1 |  |  | 10 | 0.693 | 0.1716 | Yes |
| 12 | POSTN |  |  | 11 | 0.689 | 0.1849 | Yes |
| 13 | PMEPA1 |  |  | 12 | 0.687 | 0.1982 | Yes |
| 14 | TXN |  |  | 13 | 0.686 | 0.2115 | Yes |
| 15 | PKM |  |  | 14 | 0.676 | 0.2247 | Yes |
| 16 | IGFBP3 |  |  | 15 | 0.664 | 0.2375 | Yes |
| 17 | DKK1 |  |  | 16 | 0.656 | 0.2502 | Yes |
| 18 | SPP1 |  |  | 17 | 0.642 | 0.2627 | Yes |
| 19 | CAVIN1 |  |  | 18 | 0.641 | 0.2751 | Yes |
| 20 | UBE2S |  |  | 19 | 0.634 | 0.2874 | Yes |
| 21 | S100A13 |  |  | 20 | 0.627 | 0.2996 | Yes |
| 22 | COTL1 |  |  | 21 | 0.624 | 0.3117 | Yes |
| 23 | TPM4 |  |  | 22 | 0.623 | 0.3238 | Yes |
| 24 | SMS |  |  | 23 | 0.619 | 0.3358 | Yes |
| 25 | TUBA1C |  |  | 24 | 0.619 | 0.3478 | Yes |
| 26 | SH3BGRL3 |  |  | 25 | 0.618 | 0.3598 | Yes |
| 27 | PFN1 |  |  | 26 | 0.591 | 0.3712 | Yes |
| 28 | S100A16 |  |  | 27 | 0.587 | 0.3826 | Yes |
| 29 | CSRP2 |  |  | 28 | 0.574 | 0.3937 | Yes |
| 30 | CCND1 |  |  | 29 | 0.566 | 0.4047 | Yes |
| 31 | ANXA1 |  |  | 30 | 0.564 | 0.4157 | Yes |
| 32 | PTHLH |  |  | 31 | 0.556 | 0.4265 | Yes |
| 33 | MAP3K7CL |  |  | 32 | 0.546 | 0.4370 | Yes |
| 34 | S100A10 |  |  | 33 | 0.540 | 0.4475 | Yes |
| 35 | CAV1 |  |  | 34 | 0.536 | 0.4579 | Yes |
| 36 | CAST |  |  | 35 | 0.534 | 0.4682 | Yes |
| 37 | TMEFF2 |  |  | 36 | 0.526 | 0.4785 | Yes |
| 38 | UPP1 |  |  | 37 | 0.523 | 0.4886 | Yes |
| 39 | CCDC85B |  |  | 38 | 0.523 | 0.4987 | Yes |
| 40 | FBXO32 |  |  | 39 | 0.523 | 0.5089 | Yes |
| 41 | PTTG1 |  |  | 40 | 0.520 | 0.5189 | Yes |
| 42 | NETO2 |  |  | 41 | 0.517 | 0.5290 | Yes |
| 43 | VIPR1 |  |  | 42 | 0.501 | 0.5387 | Yes |
| 44 | CTSC |  |  | 43 | 0.498 | 0.5483 | Yes |
| 45 | SRM |  |  | 44 | 0.493 | 0.5579 | Yes |
| 46 | CTSV |  |  | 45 | 0.491 | 0.5674 | Yes |
| 47 | DRAP1 |  |  | 46 | 0.485 | 0.5768 | Yes |
| 48 | STK17A |  |  | 47 | 0.483 | 0.5862 | Yes |
| 49 | ANXA2 |  |  | 48 | 0.482 | 0.5956 | Yes |
| 50 | CTNNBIP1 |  |  | 49 | 0.480 | 0.6049 | Yes |
| 51 | SLC14A1 |  |  | 50 | 0.480 | 0.6142 | Yes |
| 52 | S100A11 |  |  | 51 | 0.475 | 0.6234 | Yes |
| 53 | TPI1 |  |  | 52 | 0.473 | 0.6326 | Yes |
| 54 | TMSB10 |  |  | 53 | 0.473 | 0.6417 | Yes |
| 55 | ANK3 |  |  | 54 | 0.473 | 0.6509 | Yes |
| 56 | SPATS2L |  |  | 55 | 0.464 | 0.6599 | Yes |
| 57 | SYNJ2 |  |  | 56 | 0.461 | 0.6688 | Yes |
| 58 | HOXB2 |  |  | 57 | 0.455 | 0.6777 | Yes |
| 59 | CDC42EP3 |  |  | 58 | 0.450 | 0.6864 | Yes |
| 60 | PLP2 |  |  | 59 | 0.446 | 0.6950 | Yes |
| 61 | EIF6 |  |  | 60 | 0.445 | 0.7036 | Yes |
| 62 | OAZ1 |  |  | 61 | 0.444 | 0.7123 | Yes |
| 63 | DCBLD2 |  |  | 62 | 0.444 | 0.7209 | Yes |
| 64 | WDR1 |  |  | 63 | 0.439 | 0.7294 | Yes |
| 65 | GAP43 |  |  | 64 | 0.438 | 0.7379 | Yes |
| 66 | CAPN2 |  |  | 65 | 0.434 | 0.7463 | Yes |
| 67 | C12orf75 |  |  | 66 | 0.432 | 0.7547 | Yes |
| 68 | TUBB4B |  |  | 67 | 0.429 | 0.7630 | Yes |
| 69 | MYL6 |  |  | 68 | 0.420 | 0.7711 | Yes |
| 70 | PPP1R14B |  |  | 69 | 0.418 | 0.7793 | Yes |
| 71 | DSTN |  |  | 70 | 0.417 | 0.7874 | Yes |
| 72 | RPSA |  |  | 71 | 0.415 | 0.7954 | Yes |
| 73 | MMP24OS |  |  | 72 | 0.411 | 0.8034 | Yes |
| 74 | RPS12 |  |  | 73 | 0.411 | 0.8114 | Yes |
| 75 | DPYSL3 |  |  | 74 | 0.411 | 0.8193 | Yes |
| 76 | PLIN3 |  |  | 75 | 0.410 | 0.8273 | Yes |
| 77 | CLIC1 |  |  | 76 | 0.406 | 0.8352 | Yes |
| 78 | PRELID1 |  |  | 77 | 0.406 | 0.8430 | Yes |
| 79 | HINT1 |  |  | 78 | 0.402 | 0.8508 | Yes |
| 80 | PRDX6 |  |  | 79 | 0.398 | 0.8586 | Yes |
| 81 | LDHA |  |  | 80 | 0.394 | 0.8662 | Yes |
| 82 | TWF2 |  |  | 81 | 0.391 | 0.8738 | Yes |
| 83 | USP53 |  |  | 82 | 0.390 | 0.8813 | Yes |
| 84 | PHLDA1 |  |  | 83 | 0.389 | 0.8889 | Yes |
| 85 | PYGB |  |  | 84 | 0.388 | 0.8964 | Yes |
| 86 | ARL4C |  |  | 85 | 0.386 | 0.9039 | Yes |
| 87 | ACTB |  |  | 86 | 0.386 | 0.9114 | Yes |
| 88 | ARPC1A |  |  | 87 | 0.385 | 0.9188 | Yes |
| 89 | IGFBP5 |  |  | 88 | 0.385 | 0.9263 | Yes |
| 90 | PDLIM4 |  |  | 89 | 0.385 | 0.9338 | Yes |
| 91 | TKT |  |  | 90 | 0.384 | 0.9412 | Yes |
| 92 | GADD45GIP1 |  |  | 91 | 0.383 | 0.9486 | Yes |
| 93 | RPL8 |  |  | 92 | 0.381 | 0.9560 | Yes |
| 94 | PMAIP1 |  |  | 93 | 0.380 | 0.9634 | Yes |
| 95 | ACTG1 |  |  | 94 | 0.380 | 0.9708 | Yes |
| 96 | AP1S2 |  |  | 95 | 0.379 | 0.9781 | Yes |
| 97 | ARPC5 |  |  | 96 | 0.377 | 0.9854 | Yes |
| 98 | TXNRD1 |  |  | 97 | 0.376 | 0.9927 | Yes |
| 99 | TWIST1 |  |  | 98 | 0.376 | 1.0000 | Yes |
Table: GSEA details [plain text format]

  

Fig 2: LARSSON\_STATE\_1: Random ES distribution      
 Gene set null distribution of ES for **LARSSON\_STATE\_1**
